# Cleaning and retreatment protocol for a debonded ceramic restoration

**DOI:** 10.4317/jced.51891

**Published:** 2015-02-01

**Authors:** Juan-Luis Román-Rodríguez, Jorge Alonso-Pérez-Barquero, August Bruguera-Álvarez, Rubén Agustín-Panadero, Antonio Fons-Font

**Affiliations:** 1 DDS, MSc, PhD, Associate Lecturer, Department of Dental medicine, Prosthodontic and Occlusion Teaching Unit, University of Valencia, University of Valencia General Studies (UVGS) Spain; 2 DDS, Lecturer in Prosthodontics. Prosthodontic and Occlusion Teaching Unit, UVGS, Spain; 3 CDT, Dental Technician, Dental Design Laboratory, Barcelona, Spain; 4 DDS, PhD, MD, Senior Lecturer, Department of Dental medicine, Prosthodontic and Occlusion Teaching Unit, UVGS, Spain

## Abstract

Objectives: The aim of this article is to propose a resin cement cleaning protocol for use before recementing a debonded restoration. Study Design: Ceramic samples were fabricated from IPS d.sign® and IPS e.max Press® and were treated with hydrofluoric acid etching (HF), or HF+silane (S), or HF+S+adhesive or HF+S+A+resin cement. All samples were placed in a furnace at 650º for one minute in order to attempt to pyrolyze the composite. Each step was examined under scanning electron microscopy (SEM). Results: When the cleaning protocol had been performed, it left a clean and retentive surface. Conclusions: If the restoration is placed in a furnace at 650º for one minute, the composite cement will burn or pyrolyze and disappear, allowing conventional retreatment of the ceramic before rebonding.

** Key words:**Ceramic, debond, surface treatment.

## Introduction

**Figure 1:**
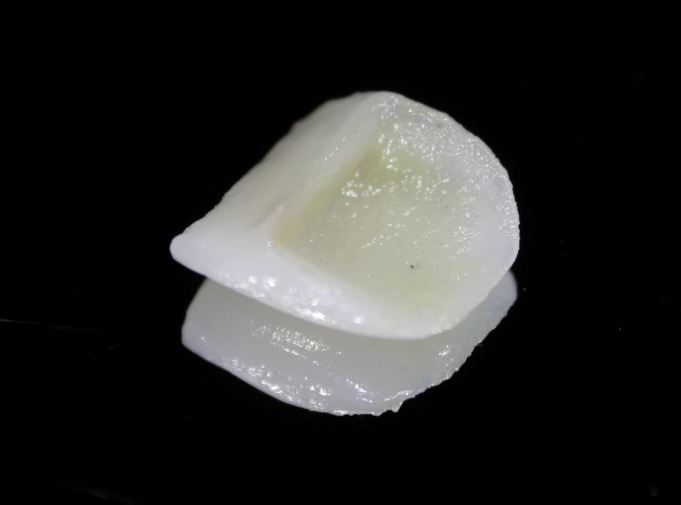
Debonded ceramic restoration.

Cementing ceramic restorations requires a bonding technique and a resin luting cement. If the restoration debonds, the remains of the adhesive must be eliminated from the restoration before repeating the conventional bond procedure: conditioning with hydrofluoric acid, application of a silane coupling agent, adhesive and resin cement. The debond rate of ceramic restorations may be as high as 9% (1). When a restoration debonds, the resin cement almost always remains adhered to it (2) (Fig. 1). This must be removed before the restoration can be recemented (3,4). The aim of this article is to propose a resin cement cleaning protocol for use before recementing a debonded restoration.

## Material and Methods

Ceramic samples (n=10) were fabricated (3x3x1mm) from IPS d.sign® and IPS e.max Press®, (Ivoclar Vivadent, Schaan, Liech-tenstein). Two samples were excluded from surface treatment. One sample of each ceramic was treated with hydrofluoric acid etching (HF), another pair with HF+silane (S), one pair with HF+S+adhesive and the last pair with HF+S+A+resin cement. All samples were placed in a furnace at 650º for one minute in order to attempt to pyrolyze the composite. Each step was examined under scanning electron microscopy (SEM).

## Results

**Figure 2:**
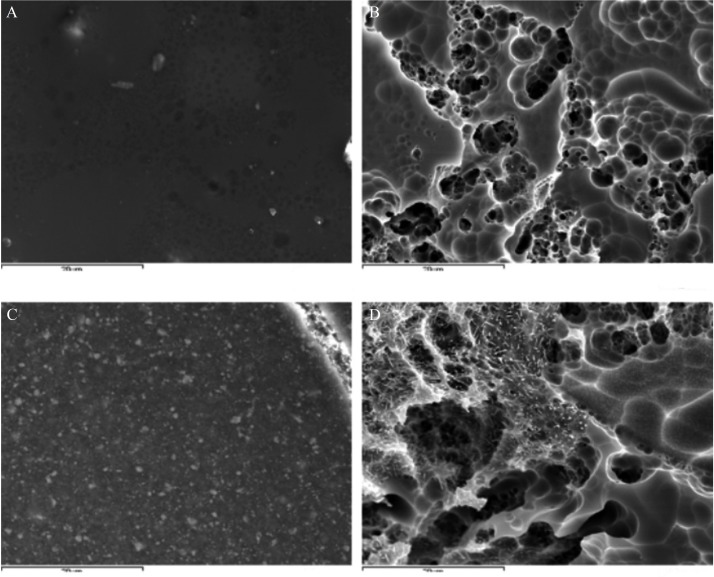
A) IPS d.sign (2500x); B) IPS d.sign HF 9.6% for 2 minutes (2500x); C) After silane coupling agent, adhesive and resin cement application (2500x); D) Placed in furnace for 1 minute and then etched with 9.6% HF for 2 minutes (2500x).

**Figure 3:**
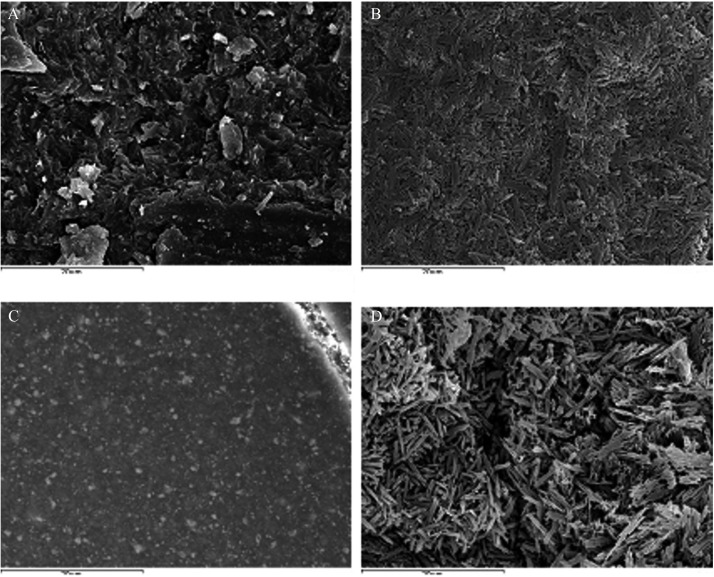
A) IPS e.max Press (2500x); B) IPS e.max Press + 4.9% HF for 20 seconds (2500x); C) Composite cement surface (2500x); D) When the cement has been burned away it is re-etched with 4.9% HF for 20 seconds (2500x).

Etching with HF brought about a change to the ceramic surface, which passed from having an almost completely smooth surface texture (Fig. 2a) or irregular pressed surface (Fig. 3a) to a highly anfractuous and retentive surface, with numerous hollows into which the resin cement could penetrate and so remain retained when set (Figs. 2b, 3b). After applying silane, adhesive and cement, the surface appeared smooth and only the resin remained visible (Figs. 2c, 3c). When the cleaning protocol had been performed, it left a clean and retentive surface (Figs. 2d, 3d).

## Discussion

The two ceramic surfaces investigated differed as a result of their fabrication techniques (5) (IPS d.sign: layering technique; IPS e.max Press: pressure injection) (Figs. 2a, 3a). HF etching produced different patterns due to the two ceramics’ different compositions, (5) although in both cases a highly retentive surface was produced for bonding (Figs. 2b, 3b). This was eliminated after heating in the furnace because the composite’s melting point is lower than that of ceramic.

A ceramic restoration that has debonded must be placed in the furnace at 650º for one minute to burn away the remaining resin cement before conventional mechanical (HF) and chemical (silane, adhesive and cement) treatment is performed. In this way, the restoration can be rebonded obtaining maximum ceramic adhesion. ( Table 1).

**Table 1:**
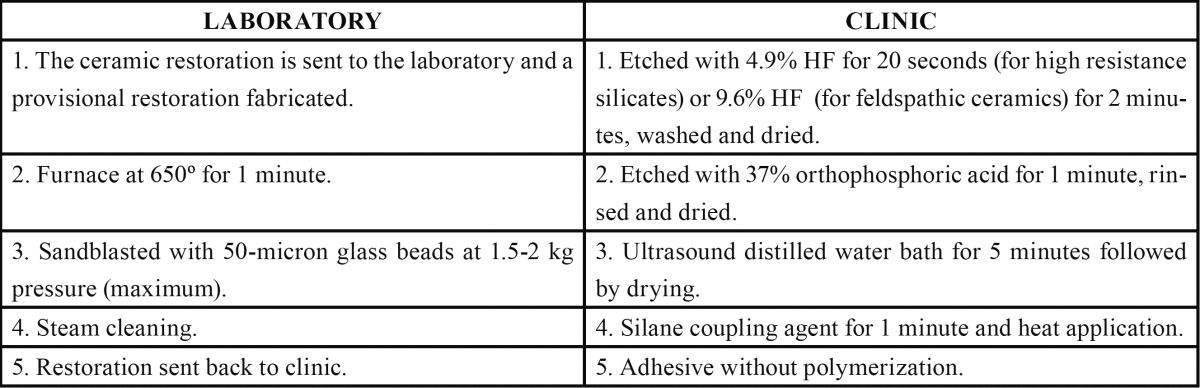
Cleaning and retreatment protocol for debonded ceramic restoration.

.
